# Recovery of divergent avian bornaviruses from cases of proventricular dilatation disease: Identification of a candidate etiologic agent

**DOI:** 10.1186/1743-422X-5-88

**Published:** 2008-07-31

**Authors:** Amy L Kistler, Ady Gancz, Susan Clubb, Peter Skewes-Cox, Kael Fischer, Katherine Sorber, Charles Y Chiu, Avishai Lublin, Sara Mechani, Yigal Farnoushi, Alexander Greninger, Christopher C Wen, Scott B Karlene, Don Ganem, Joseph L DeRisi

**Affiliations:** 1 Departments of Biochemistry, Microbiology and Medicine, Howard Hughes Medical Institute and University of California, San Francisco, 94143, USA; 2 The Exotic Clinic, Herzlyia, 46875, Israel; 3 Rainforest Clinic for Birds and Exotics, Loxahatchee, FL, 33470, USA; 4 Division of Infectious Diseases, University of California, San Francisco, 94143, USA; 5 Division of Avian & Fish Diseases, Kimron Veterinary Institute, Bet Dagan, 50250, Israel; 6 Biological and Medical Informatics Program, University of California, San Francisco, 94143, USA; 7 Lahser Interspecies Research Foundation, Bloomfield Hills, MI, 48302, USA

## Abstract

**Background:**

Proventricular dilatation disease (PDD) is a fatal disorder threatening domesticated and wild psittacine birds worldwide. It is characterized by lymphoplasmacytic infiltration of the ganglia of the central and peripheral nervous system, leading to central nervous system disorders as well as disordered enteric motility and associated wasting. For almost 40 years, a viral etiology for PDD has been suspected, but to date no candidate etiologic agent has been reproducibly linked to the disease.

**Results:**

Analysis of 2 PDD case-control series collected independently on different continents using a pan-viral microarray revealed a bornavirus hybridization signature in 62.5% of the PDD cases (5/8) and none of the controls (0/8). Ultra high throughput sequencing was utilized to recover the complete viral genome sequence from one of the virus-positive PDD cases. This revealed a bornavirus-like genome organization for this agent with a high degree of sequence divergence from all prior bornavirus isolates. We propose the name avian bornavirus (ABV) for this agent. Further specific ABV PCR analysis of an additional set of independently collected PDD cases and controls yielded a significant difference in ABV detection rate among PDD cases (71%, n = 7) compared to controls (0%, n = 14) (P = 0.01; Fisher's Exact Test). Partial sequence analysis of a total of 16 ABV isolates we have now recovered from these and an additional set of cases reveals at least 5 distinct ABV genetic subgroups.

**Conclusion:**

These studies clearly demonstrate the existence of an avian reservoir of remarkably diverse bornaviruses and provide a compelling candidate in the search for an etiologic agent of PDD.

## Background

Proventricular dilatation disease (PDD) is considered by many to be the greatest threat to aviculture of psittacine birds (parrots). This disease has been documented in multiple continents in over 50 different species of psittacines as well as captive and free-ranging species in at least 5 other orders of birds [1-5]. Most, if not all major psittacine collections throughout the world have experienced cases of PDD. It has been particularly devastating in countries like Canada and northern areas of the United States where parrots are housed primarily indoors. However, it is also problematic in warmer regions where birds are typically bred in outdoor aviaries. Moreover, captive breeding efforts for at least one psittacine which is thought to be extinct in the wild, the Spix's macaw (*Cyanopsitta spixii*), have been severely impacted by PDD.

PDD is an inflammatory disease of birds, first described in the 1970s as Macaw Wasting Disease during an outbreak among macaws (reviewed in [3]). PDD primarily affects the autonomic nerves of the upper and middle digestive tract, including the esophagus, crop, proventriculus, ventriculus, and duodenum. Microscopically, the disease is recognized by the presence of lymphoplasmacytic infiltrates within myenteric ganglia and nerves. Similar infiltrates may also be present in the brain, spinal cord, peripheral nerves, conductive tissue of the heart, smooth and cardiac muscle, and adrenal glands. Non-suppurative leiomyositis and/or myocarditis may accompany the neural lesions [6-9]. Clinically, PDD cases present with GI tract dysfunction (dysphagia, regurgitation, and passage of undigested food in feces), neurologic symptoms (e.g. ataxia, abnormal gait, proprioceptive defects), or both [3]. Although the clinical course of the disease can vary, it is generally fatal in untreated animals [3].

The cause of PDD is unknown, but several studies have raised the possibility that PDD may be caused by a viral pathogen. Evidence for an infectious etiology stems from the initial outbreaks of Macaw Wasting Disease, and other subsequent outbreaks of PDD [2,10]. Reports of pleomorphic virus-like particles of variable size (30–250 nm) observed in tissues of PDD affected birds [8] led to the proposal that paramyxovirus (PMV) may cause the disease; however, serological data has shown that PDD affected birds lack detectable antibodies against PMV of serotypes 1–4, 6, and 7, as well as against avian herpes viruses, polyomavirus, and avian encephalitis virus [3]. Similarly, a proposed role for equine encephalitis virus in PDD has been ruled out [11]. Enveloped virus-like particles of approximately 80 nm in diameter derived from the feces of affected birds have been shown to produce cytopathic effect in monolayers of macaw embryonic cells [12], but to date no reports confirming these results or identifying this possible agent have been published. Likewise, adeno-like viruses, enteroviruses, coronaviruses and reoviruses have also been sporadically documented in tissues or excretions of affected birds [3,13,14] yet in each case, follow-up evidence for reproducible isolation specifically from PDD cases or identification of these candidate agents has not been reported. Thus, the etiology of PDD has remained an open question.

To address this question, we have turned to a comprehensive, high throughput strategy to test for the presence of known or novel viruses in PDD affected birds. We employed the Virus chip, a DNA microarray containing representation of all viral taxonomy to interrogate 2 PDD case/control series independently collected on two different continents for the presence of viral pathogens. We report here the detection of a novel bornavirus signature in 62.5% of the PDD cases and none of the controls. These bornavirus-positive samples were confirmed by virus-specific PCR testing, and the complete genome sequence has been recovered by ultra-high throughput sequencing combined with conventional PCR-based cloning.

Bornaviruses are a family of negative strand RNA viruses whose prototype member is Borna Disease Virus (BDV), an agent of encephalitis whose natural reservoir is primarily horses and sheep [15]. Although experimental transmission of BDV to many species (including chicks [16]) has been described, there is little information on natural avian infection, and existing BDV isolates are remarkable for their relative sequence homogeneity. The agent reported here, which we designate avian bornavirus (ABV) is highly diverged from all previously identified members of the *Bornaviridae *family and represents the first full-length bornavirus genome cloned directly from avian tissue. Subsequent PCR screening for similar ABVs confirmed a detection rate of approximately 70% among PDD cases and none among the controls. Sequence analysis of a single complete genome and all of the additional partial sequences that we have recovered directly from the PDD case specimens suggests that the viruses detected in cases of PDD form a new, genetically diverse clade of the *Bornaviridae*.

## Results

### Microarray-based detection of a Bornaviridae signature in PDD cases

**Figure 1:**
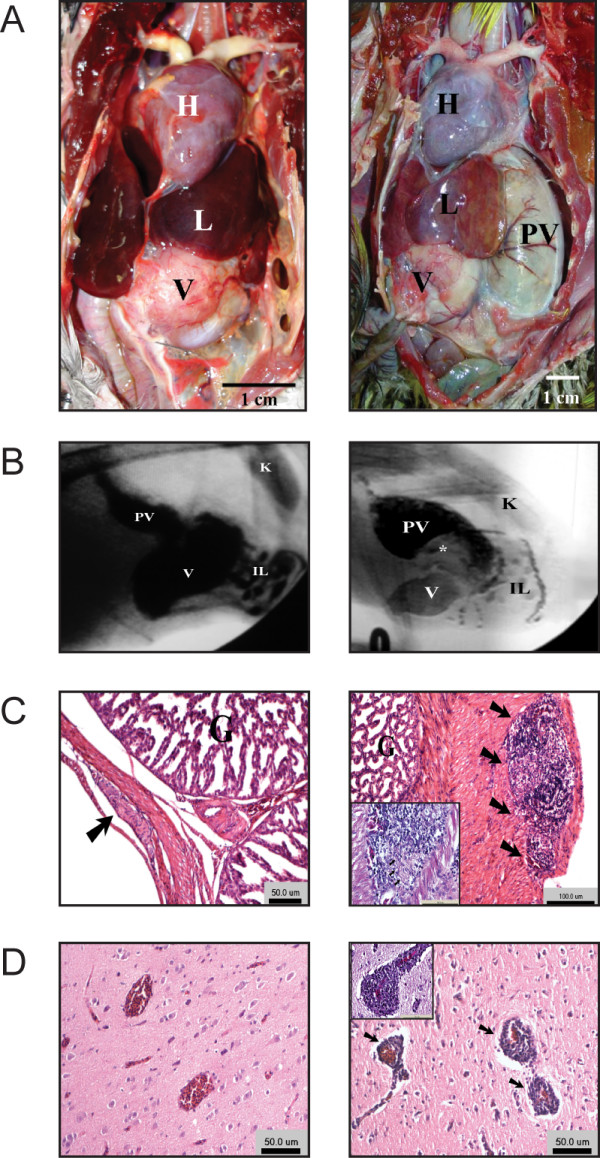
**Clinical presentation of proventricular dilatation disease (PDD) cases and controls**. A. Necropsy view of control (left panel) African gray parrot (*Psittacus erithacus*) that died of other causes. The normal-sized proventriculus is not visible in this view as it lies under the left liver lobe (L). Necropsy view of a great green macaw (*Ara ambiguus*) with PDD (right panel). The proventriculus (PV) is markedly distended and extends laterally well beyond the left lobe of L. The heart (H) is marked for orientation. B. Contrast fluoroscopy view of control (left panel) African gray parrot (*Psittacus erithacus*) 1.5 hours after administration of barium sulfate. The kidney (K) is marked for orientation. The outline of both the PV and V is clearly visible, with normal size and shape. Within the intestinal loops (IL), wider and thinner sections represent active peristalsis. Right panel, representative PDD case, Eclectus parrot (*Eclectus roratus*) 18 hours after administration of barium. The PV is markedly distended and contains most of the contrast material, with less in the V and within the IL. A large filling defect (*) representing impacted food material. The kidney (K) is shown for orientation. These findings are typical for PDD; however PDD was not confirmed by histology in this case. C. Proventriculus histopathology. Hematoxylin and eosin staining of proventriculus histological sections from a blue and yellow macaw (*Ara ararauna*) with PDD. Proventricular gland (G) is shown for orientation. Left panel, normal appearing myenteric ganglion detected within the proventriculus of this case (arrow); right panel, marked lymphoplasmacytic infiltration present within a myenteric ganglion (arrows). Right panel inset, higher magnification. D. CNS histopathology. Hematoxylin and eosin staining of a cerebral section from a control (left panel) African gray parrot (*Psittacus erithacus*) that died of other causes. Right panel, African gray parrot (*Psittacus erithacus*) with PDD. Perivascular cuffing is evident around blood vessels (arrows). Inset, higher magnification.

To identify a possible viral cause of PDD, we applied the Virus chip, a DNA microarray containing 70 mer oligonucleotide probes representing all known viral sequences conserved at multiple nodes of the viral taxonomic tree [17,18] to identify viral signatures unique to histologically confirmed cases of PDD. At the outset of this study, specimens from two independently collected PDD case/control series were available for this investigation (Figure 1, Materials and Methods). The first series (n = 8), from samples originating in the United States, consisted of crop biopsy specimens from 3 histologically confirmed PDD cases and 5 controls that were provided for nucleic acid extraction and follow-up Virus chip analysis. The samples from the second series (n = 8) originated in Israel, where total RNA and DNA from proventriculus, ventriculus and brain specimens were extracted from 5 PDD cases and 3 controls. For each series, total RNA was reverse-transcribed with random primers, PCR-amplified, and fluorescently labeled and hybridized to the Virus chip microarray as previously described [18].

**Figure 2:**
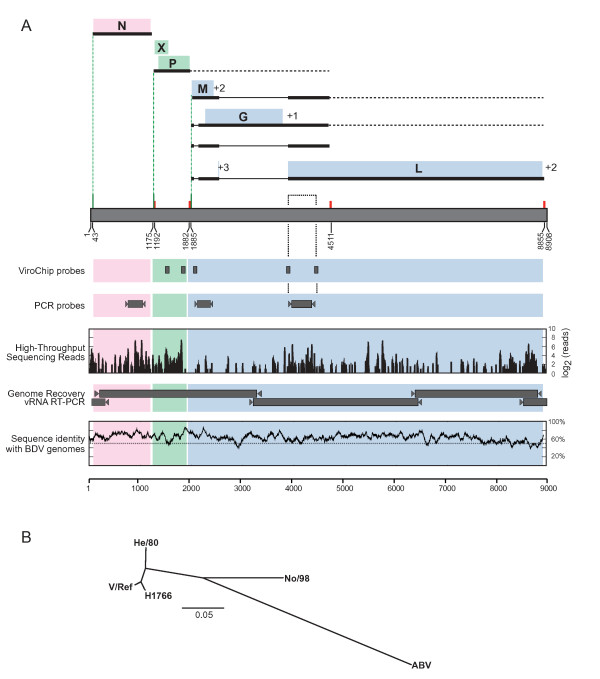
**Avian bornavirus (ABV) genome sequence recovery and comparative analysis to Borna disease virus (BDV) genomes**. A. *Bornaviridae *genome schematic. Grey bar at base, non-segmented negative sense viral RNA (vRNA) of *Bornaviridae *genome; coordinates of major sequence landmarks highlighted below. Green bars and dashed lines, transcription initiation sites (TISs); red bars, transcription termination sites. Distinct ORF-encoding transcription products and the gene products they encode are diagrammed above: TIS1 transcripts encoding nucleocapsid (N) gene, pink; TIS2 transcripts encoding phosphoprotein (P) and X genes, green; TIS3 transcripts encoding the matrix (M), glycoprotein (G) and polymerase (large or 'L') gene, blue. Exons, thick solid black lines; introns, thin solid black lines; dashed black lines, 3'ends of transcripts generated transcription termination read-through; shaded boxes, location of ORFs in transcripts; reading frames for ORFs from multiple genes generated from TIS3 indicated at right. Array probes track, *Bornaviridae *oligonucleotide 70 mer probes from the Virochip array. PCR primers track, primers generated for PCR follow up and screening of specimens in this study for detection of *Bornaviridae *species with expected product diagrammed below. vRNA RT-PCR track, overlapping vRNA clones and RACE products recovered directly from RNA extracted from crop tissue of a histologically confirmed case of PDD. Solexa reads track shows distribution of 33 mer reads with at least 15 bp sequence identity to recovered ABV genome sequence. Sequence identity with BDV genomes track shows scanning average pairwise nucleotide sequence identity (window size of 100 nucleotides, advanced in single nucleotide steps) shared between ABV and all BDV genome sequences in NCBI. A dashed line on the graph indicates 50% identity threshold for reference. B. Phylogenetic analysis of ABV genome and the 4 representative BDV genome isolates. Neighbor-joining phylogenetic trees based on nucleotide sequences of the ABV genome sequence [GenBank:EU781967] and the following representative BDV genome sequences: H1766 [GenBank:AJ311523], V/Ref [GenBank:NC_001607], He/80 [GenBank:L27077], and No/98 [GenBank:AJ311524)] Scale bar, genetic distance.

In these combined PDD case/control series, a *Bornaviridae *signature was detectable in 62.5% of the cases and none of the controls (Table 1). In the US cohort, which contained only GI tract specimens, we detected a bornavirus in 2 of 3 cases. Surprisingly, in samples from the Israeli PDD case/control series for which we had both GI tract and brain specimen RNA for each animal, we detected the *Bornaviridae *signature in 3 of the cases, but only in samples derived from brain tissue. These signatures were unambiguously confirmed by follow-up PCR and sequence recovery, using primers based on the sequences of the most strongly annealing *Bornaviridae *oligonucleotides on the microarray (Figure 2, Array probes and PCR probes tracks). These analyses revealed the presence of a set of surprisingly divergent avian bornaviruses (ABVs) in the PDD cases; the recovered sequences shared less than 70% sequence identity to any of the previously identified mammalian bornavirus isolates in the NCBI database.

**Table 1:** ABV detection in PDD

	cases^a^	controls^b^	totals
Virochip^+^	5	0	5
Virochip^-^	3	8	11
totals	8	8	16

^a^3 crop biopsies from US source and 5 brain and proventriculus/ventriculus biopsies from Israel source were examined, with ABV detected in 2 of crop specimens and 3 brain specimens. ^b^5 crop biopsies from US source and 3 brain and proventriculus/ventriculus biopsies from Israel source were examined.

### Recovery of complete genome sequence of a divergent avian bornavirus (ABV) from a PDD case via ultra high-throughput sequencing and conventional RT-PCR

To determine if the sequence fragments we detected among specimens derived from PDD cases corresponded to the presence of a full-length bornavirus, we performed unbiased deep sequencing on a PCR-confirmed bornavirus positive PDD case that contained the highest concentration of RNA. To recover both mRNA and vRNA present in the sample, RNA from this specimen was linearly amplified with both oligo(dT) and random hexamer primers, and then PCR-amplified using a modified random amplification strategy compatible with the Solexa sequencing platform (Materials and Methods). An initial set of 1.4 million 33 mer reads was obtained from this template material. Filtering on read quality, insert presence, and sequence complexity reduced this data set to 600,000 unique reads. Additional ELAND and iterative BLAST analyses ([19] Materials and Methods) of these reads against all avian sequences in NCBI (including ESTs, n = 918,511) identified reads in the dataset with at least 22 nucleotides of sequence identity likely derived from host transcripts randomly amplified during sequencing sample preparation. The 322,790 reads that passed this host filter were next screened for the presence of bornavirus sequence through similar ELAND and iterative BLAST analyses (Materials and Methods) using a database generated from all Borna Disease virus (BDV) sequences present in NCBI (n = 207) and the sequences we had recovered from PCR follow-up of the PDD samples that tested positive for bornavirus by Virus chip microarray (n = 5). These analyses provided us with 1400 reads with at least a match of 15 or more nucleotides (blastn) or 7 or more predicted amino acids (tblastx) to known BDV sequences.

Mapping these 1400 reads onto their corresponding positions on a consensus sequence for the 14 publicly available BDV genome sequences revealed spikes of high read coverage distributed discontinuously across the entire span of the BDV genome consensus. Reads containing blastn scores ≥ 90% identity to known BDV sequences were used as source sequences for primer design for PCR and sequence recovery of additional bornavirus sequence from both mRNA and vRNA templates present in the PDD specimen. Sequences recovered in this manner facilitated subsequent primer design for recovery of complete genome sequence via RT-PCR of 3 large overlapping fragments of the genome and 5'- and 3'-RACE (Figure 2A, vRNA RT-PCR track) directly from negative stranded vRNA present in the total RNA extracted from this clinical specimen.

As our initial PCR results suggested, the bornavirus genome sequence we recovered is quite diverged from all known BDV genomes, including the BDV isolate No/98, a divergent isolate sharing only 81% sequence identity with all other BDV genomes [20]. Overall, this newly recovered bornavirus genome sequence shares only 64% sequence identity at the nucleotide level to each of the complete BDV genomes. Scanning pairwise sequence identity analysis indicates this genetic divergence exists across the entire genome (Figure 2A, Sequence identity shared with BDV genomes track). Given this divergence, we re-examined the depth and distribution of the 322,790 reads from this specimen that passed the host filter to determine if we had missed reads derived from the recovered ABV in our initial screen against all BDV sequences. Not surprisingly, this retrospective BLAST analysis revealed an additional 2600 reads from across the recovered bornavirus genome that were missed in the initial BLAST analyses due to the lack of sequence conservation between the ABV sequence and the available BDV sequences (Figure 2A, Solexa reads track). In total, approximately 1% of all the high throughput shotgun reads could be mapped to the recovered bornavirus genome.

Despite this sequence divergence, this avian bornavirus genomic sequence possesses all of the hallmarks of a *Bornaviridae *family member (Figure 2A): six distinct ORFs encoding homologs of the N, X, P, M, G, and L genes are detectable. Likewise, non-coding regulatory sequence elements (the inverted terminal repeat sequences ([21], see Additional file 1: Alignment of bornavirus genomes 5' and 3' termini), the transcription initiation and termination sites ([22], see Additional file 2: Alignment of transcription initiation and termination sites in bornavirus genomes), and each of the signals for pre-mRNA splicing ([23], see Additional file 3: Alignment of splice donor and acceptor sequences in bornavirus genomes) are all conserved in sequence and location, with the exception of the splice acceptor site 3 at position 4560 that has been previously found in a subset, but not all BDV genomes [24,25]. Taken together, these data provide evidence that our analysis has uncovered a novel divergent avian bornavirus (ABV) present in cases of PDD.

Phylogenetic and pairwise sequence analyses support this conclusion. Genomic and sub-genomic phylogenetic analyses of nucleotide sequences place the recovered ABV sequence on a branch distant from representative members of the 4 distinct genetic isolates of BDV for which complete genome sequences are available (Figure 2B, see Additional file 4: Phylogenetic relationships between sub-genomic loci of ABV and representative BDV genomes). Strikingly, the ABV genome sequence segregates to a position virtually equidistant from both the set of 3 closely related BDV isolates (V/Ref, H1766, and He/80) and the divergent No/98 BDV isolate (Figure 2B). Moreover, in contrast to the previously identified divergent No/98 isolate, which retains a high level of conservation with other BDV isolates at the amino acid level, the ABV isolate also shows significant sequence divergence in the predicted amino acid sequence of every ORF in the genome (Table 2).

**Table 2:** Predicted amino acid sequence similarity between ABV, the divergent BDV-No/98 and other BDV genomes

	Average % pairwise amino acid identity (min, max)*:			
Genome locus	*ABV and BDV*	*ABV and No/98*	*BDVs*	*No/98 and BDV*

N (nucleocapsid)	72.5 (72.5, 73.0)	72.0	98.9 (97.3, 100)	97.0
X (p10 protein)	40.7 (40.0, 41.0)	45.0	96.9 (96.2, 97.8)	80.6 (80.0, 81.0)
P (phosphoprotein)	59.9 (59.0, 61.0)	61.0	98.9 (98.6, 99.2)	96.8 (96.0. 97.0)
M (matrix)	84.0	84.0	98.2 (97.7, 99.4)	98.4 (98.0, 99.0)
G (glycoprotein)	65.8 (65.0, 66.0)	66.0	98.4 (96.3, 98.9)	93.4 (93.0, 94.0)
L (polymerase)	68.0	68.0	98.8 (98.6, 99.0)	93.0

*Values without parentheses have no deviation in % pairwise amino acid identity among compared isolates.

### PCR screening of additional PDD cases and controls suggests an association between the presence of ABV and PDD

Recovery of the complete ABV genome sequence confirmed that the microarray hybridization signature we detected accurately reflected the presence of bornaviruses in our PDD specimens. With these results in hand, we designed a set of PCR primers to perform ABV-specific PCR screening of an independent set of PDD case and control specimens to investigate the association between the presence of ABV and clinical signs and symptoms of PDD. An additional set of 21 samples derived from upper GI tract specimens (crop, proventriculus or ventriculus) from PDD cases and controls were screened for ABV sequences in a blinded fashion (Materials and Methods). For this analysis, we targeted three regions of the genome: 1) the L gene region of the genome that we used for PCR confirmation of the microarray results, (Figure 2, PCR probes track), 2) a subregion within the N gene and 3) a subregion within the M gene (Materials and Methods). PCR for glyceraldehyde 3 phosphate dehydrogenase (GAPDH) mRNA was performed in parallel with the ABV PCR on all specimens to control for integrity of RNA provided from each specimen. Of the 21 specimens analyzed, 5 were positive for ABV by PCR and confirmed by sequence recovery. Unmasking the clinical status of these samples revealed that 7 of the samples were derived from confirmed PDD cases and 14 samples were derived from PDD controls. Among the PDD cases, we found 71% (5/7) to be positive by ABV PCR (Table 3). In contrast, all PDD controls were negative by ABV PCR, and positive only for GAPDH mRNA. This PCR analysis provides an independent test of the statistical significance of the association between the presence of ABV and histologically confirmed PDD (P = 0.01, Fisher's Exact Test). Although we do not observe ABV in 100% of PDD cases in this series (see Discussion), our results nonetheless indicate a significant association of ABV with PDD.

**Table 3:** Analysis of significance of ABV detection rate in PDD

	cases	controls	totals
ABV PCR^+^	5	0	5
ABV PCR^-^	2	14	16
totals	7	14	21

P = 0.01, Fisher's Exact Test

### Additional ABV isolates identified through PCR screening

Because we applied stringent inclusion criteria for the above-described association analysis study, a number of ABV (+) and ABV (-) samples were excluded. From these materials, six additional ABV isolates were detected – 5 derived from cases considered clinically suspicious and a sixth isolate derived from a confirmed PDD case for which only GI content and liver specimens were available. Additional PCR screening of a set of 12 PDD control crop biopsy specimens provided to us unblinded again yielded solely ABV PCR (-) and GAPDH (+) results. These samples were excluded from the association analysis because we knew their clinical status prior to screening. We note that inclusion of these samples in statistical analyses would not diminish the association of ABV with known or suspected PDD.

### Sequence analysis of ABV isolates indicates at least 5 divergent isolates in this branch of the Bornaviridae family

Recovery of partial sequence from additional isolates of ABV (from the above PDD case/control specimens as well as an additional samples derived from known or suspected PDD cases (Materials and Methods)) from 3 distinct regions of the ABV genome provided the opportunity to further investigate the genetic diversity within this new branch of the *Bornaviridae*. Here, our description of results is restricted to comparison with representative members of the 4 major isolates of BDV, but virtually identical results were obtained when all available BDV sequences were analyzed.

**Figure 3:**
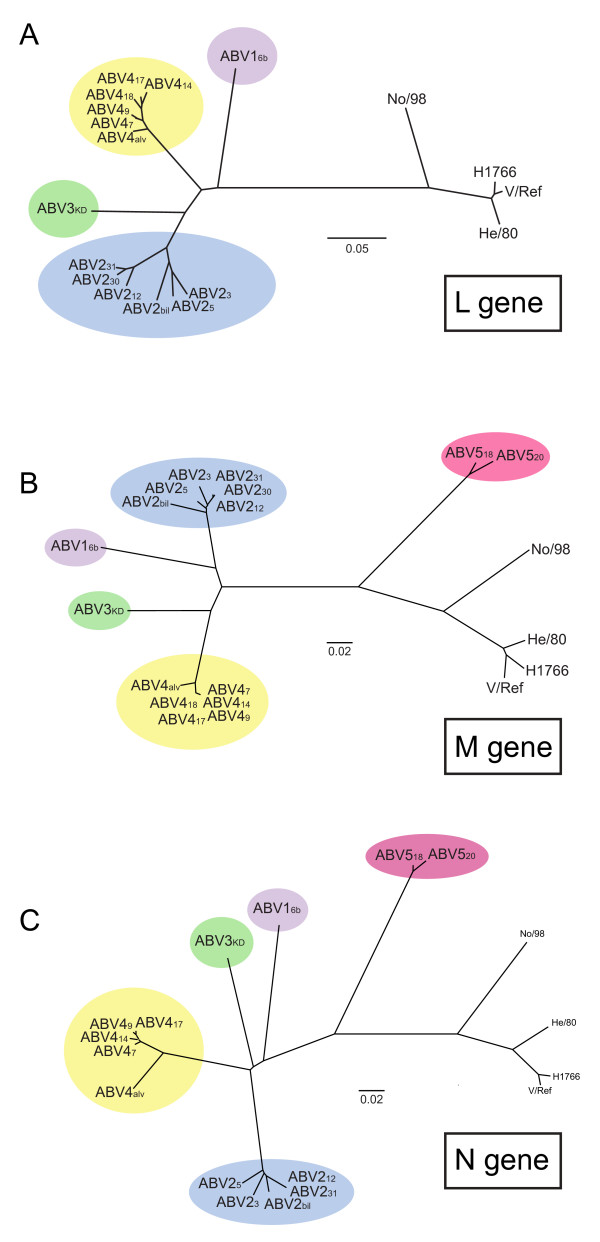
**Comparison of sequences recovered from ABV PCR screening to 4 representative genetic isolates of BDV**. Neighbor-joining Phylogenetic tree of ABV nucleotide sequences recovered by PCR screening with ABV consensus primers for subsequences within the L gene (A), the M gene (B), or the N gene (C).

As we observed for the complete ABV genome sequence, phylogenetic analysis of the recovered subgenomic ABV sequences revealed that each of the ABV isolates we recovered resides on a branch distant from the BDV isolates (Figure 3). PCR with the L gene consensus primers detected 14 isolates corresponding to 4 genetic subgroups of ABV. Each of these isolates were also detected with at least one of primer sets corresponding to the more highly expressed N gene and more conserved M gene regions of the genome; however, PCR with these two additional primer sets identified 2 additional ABV isolates that segregate to a genetically distinct 5^th ^subgroup among the ABVs (ABV5, Figure 3B and 3C). Although these 5 distinct branches correlate largely according to the geographic origin of the isolates, the genetic diversity we detect cannot be ascribed solely to differences in geographic origin of the isolates, since one of the branches (ABV4) is comprised of isolates derived from both the U.S. and Israel. Likewise, we did not detect an obvious correlation between host species and genetic subgroup of ABV among the recovered isolates.

Pairwise sequence analyses of the nucleotide and predicted amino acid sequence from the L region of the genome provide additional evidence for surprising genetic diversity among the ABV branches compared to that seen among the BDV branches (Table 4). Although derived from coding sequences of one of the more divergent genes of the bornavirus genome (Table 2, L gene), the region of the L gene we have used for PCR screening is relatively conserved among the BDV isolates, ranging from 81–98% at the nucleotide level, and 96–99% at the amino acid level (Table 4). In contrast, the sequence identity shared across this region of the genome among the ABV branches of the tree ranges from 77–83% at the nucleotide level and 86–94% at the amino acid level. Taken together with the phylogenetic analysis described above, these data provide evidence that these ABV isolates form a new, genetically diverse branch of the *Bornaviridae *phylogeny that is significantly diverged from the founder BDV isolates.

**Table 4:** Average pairwise sequence identity shared between ABV and BDV isolates*

	ABV1	ABV2	ABV3	ABV4	Ref/V	H1766	He/80	No/98
ABV1	100	**77**	**79**	**79**	**61**	**61**	**61**	**62**
ABV2	86	100	**80**	**78**	**59**	**59**	**58**	**60**
ABV3	89	89	100	**83**	**59**	**59**	**58**	**58**
ABV4	87	87	94	100	**61**	**60**	**60**	**59**
Ref/V	68	64	64	67	100	**98**	**96**	**82**
H1766	68	64	64	67	99	100	**95**	**83**
He/80	68	64	64	67	99	99	100	**81**
No/98	67	65	63	67	97	96	96	100

PCR fragment examined corresponds to bp 3735–4263 of antigenomic strand of BDV V/Ref genome isolate [GenBank:NC_001607]. Bold text, average % nucleotide identity; plain text, average % predicted amino acid identity. ABV1 isolate [GenBank:EU781953], ABV2 isolates [GenBank:EU781954 and GenBank:EU781962–EU781966], ABV3 isolate [GenBank:EU781955], ABV4 isolates [GenBank:EU781956–EU781961], Ref/V isolates [GeneBank:NC_001607, GenBank:AJ311521, GenBank:U04608], H1766 isolates GenBank:AJ311523, GenBank:AB258389, GenBank:AB246670], He/80 isolates [GenBank:L27077, GenBan:AJ311522, GenBank:AY05791, GenBank:AY114163, GenBank:AY114162, GenBank:AY114161], No/98 isolate [GenBank:AJ311524].

## Discussion

It has been almost 40 years since the first description of PDD. Although a viral etiology has long been suspected, a convincing lead for a responsible viral pathogen has been lacking. By combining veterinary clinical investigation with genomics and molecular biology, we have identified a genetically diverse set of novel avian bornaviruses (ABVs) that are likely to play a significant role in this disease. Through microarray analysis and follow-up PCR, we detected ABV sequences in 62.5% of the PDD cases in a set of specimens from two carefully collected PDD case/control series originating from two different continents. We confirmed that these assays faithfully reflect the presence of full-length bornavirus in ABV PCR positive specimens through cloning of the complete ABV vRNA sequence directly from RNA extracted from one of these ABV PCR positive PDD case specimens. We next found evidence for a significant association between the presence of ABV and clinically confirmed PDD in follow-up blinded PCR screening of a set of additional PDD cases and controls, with ABV was detected in 71% of PDD cases and none of the controls (P = 0.01, Fisher's Exact Test).

Almost all prior sightings of bornaviruses in nature have been among mammals, and the mammalian isolates have been remarkably homogeneous at the sequence level (Table 2 and [15]). The latter is a surprising feature for RNA viruses, whose RNA-dependent RNA polymerases typically have high error rates. By contrast, the ABV isolates reported here are quite diverged from their mammalian counterparts, and show substantial heterogeneity among themselves. We note with interest that a single earlier report suggesting a potential avian reservoir for bornaviruses has been presented [26]. In that study, RT-PCR based on mammalian BDV sequences was used to recover partial sequences from stool collected near duck ponds where wild waterfowl congregate. However, the resulting sequences shared ca. 98% amino acid sequence homology to the mammalian BDVs, raising the possibility that these putative avian sequences might have resulted from possible environmental or laboratory contamination [15]. Our ABV isolates, which are unequivocally of avian origin, are clearly very different from these sequences; it remains to be seen if other wild birds can indeed harbor BDV-like agents. The expanded sequence diversity of the bornaviruses discovered here should facilitate design of PCR primers that will enable expanded detection of diverse bornaviral types in future epidemiological studies.

The known neurotropism of bornaviruses makes them attractive and biologically plausible candidate etiologic agents in PDD, since (i) PDD cases have well-described neurological symptoms such as ataxia, proproceptive defects and motor abnormalities; and (ii) the central GI tract pathology in the disorder results from inflammation and destruction of the myenteric ganglia that control peristaltic activity. However, despite our success in ABV detection in PDD, we did not observe ABV in every PDD case analyzed. There are several possible explanations for this result. First, we do not know the tissue distribution (tropism) of ABV infection, or how viral copy number may vary at different sites as a function of the stage of the disease. By weighting our sample collection towards clinically overt PDD, we may have biased specimen accrual towards advanced disease. At this stage, where destruction of myenteric ganglial elements is often extensive, loss of infected cells may have contributed to detection difficulties (We note with interest in this context that in one of our case collections from Israel, virus detection occurred preferentially in CNS rather than in GI specimens). There are many precedents for such temporal variation in clinical virology – for example, in chronic hepatitis B viral loads typically decline by several orders of magnitude over the long natural history of the infection [27]. It is also possible that our detection rate may merely reflect suboptimal selection of PCR primers employed for screening; after all, our consensus primer selection was based on sequences we had recovered (L gene consensus primers) or sequence homology between the first fully sequenced ABV genome we recovered and a set of highly related mammalian BDV genome sequences (N and M gene consensus primers). We now recognize that there is substantial sequence variation within the ABVs (see Fig. 3); as more sequence diversity is recognized, better choices for more highly conserved primers will become apparent and could impact upon these prevalence estimates.

Finally, there could actually be multiple etiologic agents in PDD, with ABV infection accounting for only ~70% of the cases. Certainly both human and veterinary medicine are replete with examples of multiple agents that can trigger the same clinical syndrome – for example, at least 5 genetically unrelated viruses (hepatitis viruses A-E) are associated with acute hepatitis, and at least 3 of these can be implicated in chronic liver injury; similarly, several agents (RSV, rhinoviruses and occasionally influenza viruses) are implicated in bronchiolitis. To investigate this possibility, further high-throughput sequencing analysis of PDD cases that were negative for bornaviruses by PCR screening is currently underway.

Although ABV is clearly a leading candidate etiologic agent in PDD, formally establishing a causal role for ABV in PDD will require further experimentation. Such experiments could include (i) attempts to satisfy Koch's postulates via cultivation of ABV, followed by experimental transmission of infection and disease in inoculees, (ii) examination of seroprevalence rates in flocks with high and low PDD incidences, (iii) documentation of seroconversion accompanying development of PDD-like illnesses and (iv) examination of PDD cases by immunohistochemistry or in situ hybridization for evidence of colocalization of ABV infection at sites of histopathology. The recovery and characterization of a complete ABV genome and multiple isolates from this diverse new branch of the *Bornaviridae *family now opens the door to such investigations.

## Conclusion

By combining clinical veterinary medical investigation with comprehensive pan-viral microarray and high throughput sequence analyses, we have identified a highly diverged set of avian bornaviruses directly from tissues of PDD cases, but not controls. These results are significant for a number of reasons. First, they provide a compelling lead in the long-standing search for a viral etiology of PDD, and pave the way for further investigations to assess the link between ABV and PDD. Second, these results also unambiguously demonstrate the existence of an avian reservoir of bornaviruses, expanding our understanding of the bornavirus host range. Finally, these results also provide the first evidence that the *Bornaviridae *family is not confined to a set of genetically homogeneous species as was previously thought, but actually encompasses a set of heretofore unanticipated genetically diverse viral species.

## Methods

### PDD case and control definitions, specimen collection, and RNA extraction for pan-viral microarray screening

Two independent sets of PDD case and control specimens collected from two distinct geographic locations were independently prepared for pan-viral microarray screening and subsequent PCR screening. Sampling collection and inclusion criteria for each set are described below. Detailed information on each sample, along with results from histology, microarray, and PCR assays are provided in Additional file 5: Summary of clinical and molecular data for specimens provided in this study.

#### United States PDD case/control series

##### Specimen collection

All specimens provided for initial screening were crop tissue biopsies obtained from live psittacine birds to be used as normal controls or multiple tissue samples collected from clinically diseased birds at the time of euthanasia. Specimens were collected from client-owned birds from approximately August 2006 to May 2008 (All samples collected by S. Clubb). All of these samples originated from the southeast region of Florida. Crop biopsy tissue was collected from live birds under isoflurane anaesthesia. Following routine surgical preparation and sterile technique, the skin was incised over the center of the crop. The crop tissue was exposed and a section of tissue removed taking care to include large visible blood vessels. Fresh crop biopsy tissue was trimmed into tissue slices < 5 mm thick and submersed in RNAl*ater *(Qiagen, Inc., USA, Valencia, CA) solution immediately upon harvest and frozen within 2 minutes of collection at -20°C to -80°C according to manufacturer's protocol, and held in this manner until shipped. A duplicate sample was fixed in 10% buffered formalin for routine histological examination with H & E stain. Time of frozen storage varied (2 weeks to 12 months) as samples were accumulated prior to shipping frozen. Clinically affected birds submitted as positives were euthanized under isoflurane anaesthesia and mixed tissues (proventriculus, ventriculus, heart, liver, spleen, kidneys, brain) were placed into RNAlater within 1 minute of death and frozen within 2 minutes of death. Duplicate samples were collected for histopathologic diagnosis of PDD.

##### Inclusion criteria

PDD-positive cases were required to meet the following criteria 1) Clinical history of characteristic wasting/malabsorption syndrome with dilation of the proventriculus and/or ventriculus and presence of undigested food in the stool and in most cases, a clinical history of ataxia or other CNS signs consistent with clinical PDD, and 2) histopathology confirming the presence of moderate to extensive lymphoplasmacytic ganglioneuritis affecting crop tissue and at least one of the following additional areas: proventriculus, ventriculus, brain, adrenal gland, or myocardium. PDD-negative controls were required to be from birds with no evidence of lymphoplasmacytic neurogangliitis on histopathology derived either from 1) normal birds with no clinical history of PDD or no known exposure to PDD or 2) birds which died of other causes. Crop biopsies from samples from living birds classified as suspicious cases were also submitted. Suspicious cases were defined histologically as having lymphocytes and plasma cells surrounding neurons but not infiltrating into the neurons. An additional specimen derived from a live bird raised with two necropsy-confirmed PDD birds in Virginia was also collected for analysis. Here, only cloacal swab and blood specimens were available and the lack of histopathological confirmation and crop tissue excluded this specimen from the ABV-PDD association analysis. However, we did perform ABV PCR on these clinically suspicious specimens and the resulting viral sequences isolated were included in the subsequent comparative sequence analyses.

##### RNA extractions

For RNA extractions, specimens were thawed in RNALater, sliced into 0.5 mm × 0.5 mm pieces, transferred to 2 ml of RNABee solution (Tel-Test, Inc., Friendswood, TX), homogenized with freeze thawing and scapel mincing, then extracted in the presence of chloroform according to manufacterer's instructions. Resulting RNA was next incubated with DNase (DNA-free, Applied Biosystems/Ambion, Austin, TX) to remove any potential contaminating DNA present in the specimen.

#### Israeli case/control series

##### Specimen collection

Tissue samples were obtained from psittacine birds submitted to the Division of Avian and Fish Diseases, Kimron Veterinary Institute (KVI) Bet Dagan, Israel, for diagnostic necropsy between July 2004 and March 2008. A few additional specimens were obtained through private veterinarians. Some tissues were kept for nearly 4 years frozen either at -20°C or -80°C prior to testing, while others were fresh tissues from recent cases. The types of banked frozen tissue varied from case to case, while for some of the older cases only gastrointestinal content had been banked. Clinical histories for these birds were available from the submission forms or through communication with the submitting veterinarians. The results of ancillary tests performed at the KVI were available through the KVI computerized records.

##### Inclusion criteria

Only cases for which appropriate histological sections were available for inspection were considered for this study. These had to include brain and at least two of the following tissues: crop, proventriculus, ventriculus. The tissue-types examined for each bird for which specimens were provided are listed in Data File S1. PDD-positive cases were required to have evidence of lymphoplasmacytic infiltration of myenteric nerves and/or ganglia within one or more of the upper GI tract tissues mentioned above. These were all derived from birds that had been suspected to have PDD based on their clinical case histories and/or necropsy findings. PDD-negative controls had no detectable lesions and no evidence of non-suppurative encephalitis. For most birds in the PDD-negative group, a cause of death (other than PDD) has been determined. Two birds that came from a known PDD outbreak, but showed only cerebral lymphoplasmacytic perivascular cuffing, were classified as 'suspicious'. These were excluded from the statistical analysis, as were all other birds for which a PDD status could not be clearly determined and classified as 'inconclusive' (e.g. due to poor tissue preservation, poor section quality, or scarcity of myenteric nerves within the tissues examined).

##### RNA extraction

When possible, a sample of brain as well as a combined proventricular/ventricular sample was prepared for RNA extraction for each bird. If not available, other tissues and/or gastrointestinal content were used (see Additional file 5: Summary of clinical and molecular data for specimens provided in this study). Frozen samples were allowed to thaw for 1–2 hours at room temperature prior to handling. Then, under a laminar flow biohazard hood and using aseptic technique, approximately 1 cm^3 ^of each tissue was macerated by two passages through a 2.5 ml sterile syringe and transferred into sterile test tubes containing 4 ml nuclease-free PBS. The content of the tubes was mixed by vortex for 30 sec, and the tubes were placed overnight at 4°C. RNA extraction was performed on the following day, using either the QIAamp^® ^viral RNA kit (Qiagen, Valencia, CA; batch1&2, specimens 1–8) or the TRI Reagent^® ^kit (Molecular Research Center, Cincinnati, OH; all other specimens), following the manufacturers' instructions. The end product was either provided lyophilized (batches 1 and 2, samples 1–9) as a dry pellet, or re-suspended in 40 ul nuclease-free water.

### Virus chip hybridization experiments

Microarray analysis of specimens was carried out as previously described [18]. Briefly, 50–200 ng of DNAse-treated total RNA from each sample was amplified and labelled using a random-primed amplification protocol and hybridized to the Virochip. Microarrays (NCBI GEO platform GPL3429) were scanned with an Axon 4000B scanner (Axon Instruments). Virochip results were analyzed using E-Predict [28] and vTaxi (K. Fischer et al., in preparation).

### PCR primers for detection of avian bornaviruses

#### Microarray-based Bornaviridae PCR primers

Initial PCR primers were generated based on two of the 70 mer microarray probes with hybridization signal in the *Bornaviridae *positive arrays that localize to positions 3676–3745 and 4201–4270 of the Bornaviridae reference sequence [GenBank:NC_001607]. Subsequences within each of these probes (BDV_LconsensusF: 5'-CCTCGCGAGGAGGAGACGCCTC-3' and BDV_LconsensusR: 5' CTGCTCTTGGCTGTGTCTGCTGC-3'; positions 3710–3729 and 4252–4230, respectively of the NCBI *Bornaviridae *reference sequence) that are 100% conserved across the 12 other fully sequenced bornavirus genome isolates in NCBI (huP2br [GenBank:AB258389], Bo/04w [GenBank:AB246670], No/98 [GenBank:AJ311524], H1766 [GenBank:AJ311523], He/80/FR [GenBank:AJ311522], V/FR [GenBank:AJ311522], virus rescue plasmid pBRT7-HrBDVc [GenBank:AY05791], CRNP5 [GenBank:AY114163], CRP3B [GenBank:AY114162], CRP3A [GenBank:AY114161], He/80 [GenBank:L27077], and V [GenBank:U040608]) were utilized for initial follow-up PCR and sequence confirmation of microarray screening results. Briefly, 1 ul of the randomly amplified nucleic acid prepared for microarray hybridization from all specimens was utilized as template for 35 cycles of PCR, under the following conditions: 94°C, 30 seconds; 50°C, 30 seconds; 72°C, 30 seconds. Resulting PCR products were gel purified, subcloned into the TOPO TA cloning vector pCR2.1 (Invitrogen, USA, Carlsbad CA) and sequenced with M13F and M13R primers.

#### Generation of ABV consensus PCR primers

Sequences recovered from BDV_LconsensusF and BDV_LconsensusR PCR products were aligned, and an additional set of ABV consensus primers biased towards the ABV sequences were identified: ABV_LconsensusF, 5'-CGCCTCGGAAGGTGGTCGG-3' (maps to positions aligning with residues 3724–3742 of BDV reference genome) and ABV_LconsensusR, 5'-GGCAYCAYCKACTCTTRAYYGTRTCAGC-3' (maps to positions aligning with residues 4233–4257 of BDV reference genome). Using identical PCR cycling conditions as described above for the microarray-based *Bornaviridae *PCR assay, these ABV consensus primers were found to be > 100X more sensitive for ABV detection compared to BDV_LconsensusF and BDV_LconsensusR primers, and were thus utilized to re-screen the initial set of PDD case and control samples provided for microarray analysis (no additional positives identified) and all subsequently provided samples. Two additional PCR primers in the N (ABV_NconsensusF: 5'-CCHCATGAGGCTATWGATTGGATTAACG-3' and ABV_NconsensusR: 5'-GCMCGGTAGCCNGCCATTGTDGG-3') and M (ABV_MconsensusF: 5'-GGRCAAGGTAATYGTYCCTGGATGGCC-3' and ABV_PconsensusR: 5'-CCAACACCAATGTTCCGAAGMCG-3') that mapped to conserved sequences shared between the complete ABV genome sequence and the 14 other fully sequenced BDV genomes in the NCBI database were also employed for PCR screening of PDD cases and controls.

### Ultra high-throughput sequencing

#### Sample preparation and sequencing

500 ng of total RNA derived from one of the PDD case specimens was linearly amplified via modification of the MesssageAmp aRNA kit (Applied Biosystems/Ambion, Austin, TX). To ensure the amplification of both mRNA and vRNA present in the specimen, T7-tailed random nonamer was mixed in an equimolar ratio with the manufacturer-provided T7-oligo(dT) primer during the 1^st ^strand synthesis step. The resulting aRNA was next used as input for a modified version of Genomic DNA sample preparation protocol for ultra high-throughput Solexa sequencing (Illumina, Hayward, CA). 400 ng of the input aRNA was reverse-transcribed with reverse transcriptase (Clontech Laboratories, Inc., Mountain View, CA) using a random nonamer tailed with 19 bp of the Solexa Long (5'-CACGACGCTCTTCCGATCTNNNNNNNNN-3') primer sequence (Illumina, Hayward CA). Following termination of reaction, first strand cDNA products were purified from the reaction with Qiagen MinElute spin column (Qiagen USA, Valencia CA). To ensure stringent separation from primers, the MinElute eluate was then filtered through a Microcon YM30 centrifugal filter (Millipore Corp., Billerica, MA). The resulting eluate served as template for 2^nd ^strand synthesis in a standard Sequenase 2.0 (USB, Cleveland, OH) reaction primed with a random nonamer tailed with 22 bp (5'-GGCATACGA GCTCTTCCGATCTNNNNNNNNN-3') of the Solexa Short primer sequence (Illumina, Hayward CA). Double-stranded DNA products were separated from primers and very short products through a second Qiagen MinElute spin column run followed by a Microcon YM50 centrifugal filter. This eluate was used as template for 10 cycles of PCR amplification with the full length Solexa L and S primers using KlenTaq LA DNA polymerase mix (Sigma-Aldrich, St. Louis, MO). PCR product was purified from the reaction with a MinElute spin column. Following cluster generation, Solexa sequencing primer was annealed to the flow cell, and 36 cycles of single base pair extensions were performed with image capture using a 1G Genome Analyzer (Illumina, Hayward, CA). The Solexa Pipeline software suite version 0.2.2.6 (Illumina, Hayward, CA) was utilized for base calling from these images. Using software default quality filters, cycles 4–36 were deemed high quality, resulting in a total of 1.4 million 33 mer reads for downstream sequence analyses.

#### Identification of Bornaviridae reads

Reads sharing 100% identity to each other or the Solexa amplification primers were filtered, reducing our initial set of 1.4 million reads to a working set of 600,000 unique reads. In order to quickly assess the homology of this set of reads to different sequence databases, we employed an iterative strategy using ELAND (**E**fficient **L**ocal **A**lignment of **N**ucleotide **D**ata) and BLAST analyses. To filter reads from our analysis potentially derived from psittacine host tissue, the working set of reads were aligned to a database of all *Aves *sequences from NCBI (n = 918,511) using ELAND, which tolerates no more than 2 base mismatches, and discards both low quality reads and reads with low sequence complexity. Reads that did not align to the *Aves *database by ELAND analysis were next re-aligned to the *Aves *database for high stringency blastn analysis (e = 10^-7^, word size = 11), followed by progressively lower stringencies (down to e = 10-2, word size = 8), corresponding to reads containing only 22 nucleotide identities to sequences in the *Aves *database. To identify reads with some homology to *Bornaviridae *sequences in the resulting set of 322,790 host-filtered reads, we re-implemented the ELAND/iterative blastn analysis strategy (down to ≥ 15 nucleotides identity) using a database of all NCBI BDV sequences (n = 207) augmented by our previously recovered ABV sequences (n = 5). An additional iterative tblastx analysis was incorporated to capture distantly related reads that shared similarity to the known BDV sequences only at the level of predicted amino acid sequence (down to ≥ 6 amino acid identity).

### Complete ABV vRNA genome sequence recovery by RT-PCR

#### Initial genome sequence recovery

Sequences from 33 mer reads from the deep sequencing with a minimum of 91% sequence identity with known BDV sequences present in the NCBI database were utilized to generate a set of primers for additional cloning and sequence recovery by RT-PCR of both mRNA and vRNA present in the clinical specimen. In this manner, we generated a hybrid assembly derived from multiple overlapping clones and 5' RACE products encompassing the ABV genome sequence.

#### vRNA genome sequence recovery

To ensure recovery of accurate sequence across the ABV genome, especially at splice junctions and transcription initiation and termination sites, we utilized the sequence from ABV hybrid assembly to design primers for recovery of 3 overlapping products by RT-PCR directed against the vRNA present in the specimen. Aliquots of 500 ng of DNAse-treated total RNA extracted from the clinical specimen were annealed with 3 primers complementary to the predicted vRNA sequences: ABV1r, 5'-ATGACCAGGACGAGGAGATG-3' (maps to residues 8831-8812 of vRNA), ABV2r, 5'-CCTGTGAATGTCTCGTTTCTG-3' (maps to residues 5754-5733 of vRNA), and ABV3r 5-TTCTTTCAGCAACCACTGACG-3' (maps to residues 2563-2543 of vRNA). Reverse transcription was carried out at 50°C for 1 hr with SuperScriptIII (Invitrogen, Carlsbad CA) according to manufacturer's instructions. Following RNase H treatment, PCR was performed on the resulting cDNA with Phusion polymerase (NEB, Ipswich, MA) with the primers used for reverse transcription and the following primers: ABV1f: 5'-GGATCATTCCTTGATGATGTATTAGC-3', (maps to residues 5567-5589) ABV2f: 5'-CAAATGGAGAGCCTGATTGG-3' (maps to residues 2378-2397) ABV3f: 5'-AATCGGTAAGTCCAGAGTCAAGG-3' (maps to residues 155-177). All products were amplified for 35 cycles under the following conditions: 98°C, 3 minutes; 98°C, 10 seconds, 50°C, 30 seconds, 72°C 3 minutes. Resulting products were gel purified, and subcloned into the TOPO T/A cloning vector pCR2.1 after incubation with Taq polymerase and dATP for 10 minutes at 72°C. For each product, 4 independent transformants were prepared for standard dideoxy sequencing on an ABI3730 sequencer (ElimBio, Hayward CA). Forward and reverse reads spanning each clone were generated using M13F and M13R and additional overlapping primers spaced at 600–800 bp intervals across the each of the clones.

#### 5' and 3' RACE to sequence at vRNA termini

vRNA RT-PCR products containing uncapped vRNA termini were captured using the First Choice RLM RACE kit (Ambion, Austin TX) with the following modifications to the standard protocol: 1) tobacco acid phosphotase treatment was omitted, 2) a phosphorylated RNA, RNAligate, 5'-p-GUUAUCACUUUCACCC-3' (gift of J. Shock, DeRisi lab) was substituted for the 3' RNA ligation-mediated RACE primer provided in the kit and ligated to 3' ends as per manufacterer's 5' RACE protocol, and 3) in the 3' RACE reverse transcription reactions, two reverse transcription reactions were performed and carried forward in parallel: one with random decamers and one with a DNA oligo complementary to oJSmer utilized in the RNA ligation step (ligateRC, 5'-p-GGGTGAAAGTGATAAC-3'). For 5' RACE, a single round of PCR was sufficient to generate a product using the vRNA specific primer ABV5RaceOuter, 5'-CAGTCGGTTCTTGGACTTGAAGTATCTAGG-3' (maps to residues 346-317 of vRNA) and manufacturer provided outer PCR primer. For 3' RACE, nested PCR was required to recover detectable PCR product of expected size using outer PCR primers oJSmerRC and the gene specific primer ABV3RaceOuter, 5'-CCCGTCTACTGTTCTTTCGCCG-3' (maps to residues 8479-8497 of vRNA), followed by inner PCR using Tailed_RNAligateRC, 5'-AAGCAGTGGTAACAACGCAGAGTACGGGTGAAAGTGATAAC-3' and the gene specific primer, ABV3RaceInner, 5'-GCAATCCAGGAATAAGCAAGCACAAA-3' (maps to residues 8595-8620 of vRNA). Both of the RACE PCR reactions were carried out with Platinum Taq polymerase (Invitrogen, Carlsbad, CA) in 35 cycles of gradient PCR (with varying annealing temperature): 94°C, 30 seconds; 55–58°C, 30 seconds; 72°C, 30 seconds. Resulting PCR products were gel purified and subcloned into TOPO T/A cloning vector pCR 4.0. For the 5' RACE products, 7 independent transformants from 3 independently generated PCR products were subcloned and sequenced with M13F and M13R primers. For the 3' RACE products, 6 independent transformants from 4 independently generated PCR products were subcloned and sequenced with M13F and M13R primers. Terminal sequences reported here reflect the majority consensus sequence obtained from these reads.

#### Genome sequence assembly

Genome sequence assemblies from both initial genome sequence recovery and vRNA genome sequence recovery were generated using Consed, version 16.0 software [29]. All bases from the resulting vRNA genome sequence assembly are covered at least 4× with a minimum Phred value of 20.

### Blinded PCR screening of additional PDD cases and controls

Beyond the initial set of 16 specimens provided for microarray analysis, specimens from a total of 38 additional PDD cases, PDD controls, and PDD suspicious birds with varied clinical histories were provided to us blinded by our 2 collaborators (see Additional file 5: Summary of clinical and molecular data for specimens provided in this study).

#### Sample processing

For specimens provided in tissue form from the US collaborators, total RNA was extracted as described above with RNABee, DNase treated, then reverse-transcribed and PCR-amplified according to our random amplification protocol for microarray sample preparation (Materials and Methods). Specimens provided from Israel in the form of extracted RNA were similarly DNAse-treated and amplified prior to PCR screening.

#### PCR screening

1 ul of the randomly amplified material generated from these RNA samples was used as input template for ABV consensus PCRs as described above. In parallel, as an independent control for input specimen RNA integrity, PCR for glyceraldehyde 3-phosphate dehydrogenase (GAPDH) mRNA was performed on all specimens using designed based on Friedman-Einat et al [30] and *Gallu gallus *GAPDH sequence: Gg_GAPDHf: 5'-AGTCATCCCTGAGCTSAAYGG*GAAGC-3' (bp708-733 in Gallus gallus cDNA (NCBI accession NM_204305), * indicates the junction of GAPDH exon 8 and 9 spanned by this primer), Gg_GAPDHr 5'-ACCATCAAGTCCACAACACGG-3' (Spans bp 1037-1017 in Gallus gallus GAPDH cDNA (NCBI accession NM_204305), maps to GAPDH exon 12). After PCR results were tallied, clinical information on all specimens tested was unmasked. A complete accounting of ABV, GAPDH PCR results, specimen type and clinical status is provided in Additional file 5: Summary of clinical and molecular data for specimens provided in this study.

#### Sample inclusion for association analysis

To reduce potential confounding due to differences in viral detection resulting from specimen tissue source, only specimens derived from upper GI tract tissue (crop, proventriculus/ventriculus) that tested positive by GAPDH mRNA PCR were included in association analysis presented in Table 3. This consisted of a total of 21 specimens, 7 of which were derived from histologically confirmed PDD cases and 14 derived from histologically negative control specimens.

#### Samples excluded from association analysis

The remaining 17 samples were excluded from the analysis because they were either 1) GAPDH-positive or GAPDH-negative samples derived from specimen other than upper GI tract tissue (GI content, brain, liver, or intestine) or 2) derived from cases that were histologically or clinically 'suspicious', but unconfirmed PDD cases. Six additional ABV PCR positives were identified among this set of samples excluded from the statistical analyses: 1 derived from GI content from a confirmed PDD case, and 5 derived from a variety of tissues from the PDD suspicious cases.

### Phylogenetic and comparative sequence analysis

Multiple sequence alignments of complete genome sequences or partial sequences derived from PCR screening studies were generated with ClustalW [31] version 1.83. Resulting alignments were used for scanning pairwise sequence analysis (window size, 100; step size 1 nucleotide steps). Additional ClustalW alignments and neighbor-joining phylogenetic trees were generated using Mega software, version 4.0.2 [32].

## List of abbreviations

ABV: Avian bornavirus; BDV: Borna diseae virus; PDD: Proventricular dilatation disease.

## Competing interests

Sequence information obtained here has been disclosed for patenting purposes. ALK, AG, SC, PS-C, KF, KS, CYC, AL, AG, SKB, DG, and JLD were all party to this disclosure in conjunction with UCSF Office of Technology Management.

## Authors' contributions

ALK participated in the conception, design, and coordination of the study, performed specimen extraction of specimens from Florida case/control study, array analyses for both sets of PDD case/control series, follow-up PCR screening and sequencing of samples and wrote the manuscript, AG orchestrated and collected the PDD case/control specimens from Israel and coordinated the clinical and histopathology analyses, and nucleic acid extraction for samples from Israel, and participated in revising the manuscript, SC orchestrated and collected the Florida PDD case/control specimens and oversaw the clinical and histopathologic analyses of these samples from Florida, and participated in revising the manuscript, PS-C carried out filtering and iterative BLAST analysis of ultra high throughput sequence data for ABV genome sequence recovery, participated in primer design and complete genome sequence recovery, and drafting the manuscript, KF participated in array analysis, developed pipeline for ultra high throughput sequence analysis, and participated in design of filtering and iterative BLAST analysis, KS performed modified library preparation for ultra high throughput sequencing and participated in revising the manuscript, CYC performed ultra high throughput sequencing and participated in revising the manuscript, AL, SM, and YF participated in clinical evaluation, specimen collection and extraction of samples from Israel, AG participated in extraction of specimens from Florida and follow-up microarray analysis and high throughput sequencing, CCW developed additional primers for PCR follow-up studies, SBK assisted in the selection of the PDD case/control specimens from Florida and participated in review of clinical and histological status of cases and controls included in the study, DG and JLD oversaw the overall conception and design of the project and supervised all phases of its execution and the drafting and revision of the manuscript.

## Supplementary Material

Additional file 1 **Alignment of bornavirus genomes 5' and 3' termini**. Bornavirus genome organization overview diagrammed as in Figure 2. Sequences in alignments shown are complementary to vRNA sequence, genome isolate names shown at left. 3' end sequence recovered for ABV genome and other BDV genomes is shown in left panel, 5' end sequence recovered for ABV genome and other BDV genomes is shown in right panel. Accession numbers for genomes aligned: hu2Pbr [GenBank:AB258389], Bo/04w [GenBank:AB246670], H1766 [GenBank:AJ311523], Ref [GenBank:NC_001607], V [GenBank:U04608], V/FR [GenBank:AJ311521], CRNP5 [GenBank:AY114163], CRP3B [GenBank:AY114162], CRP3A [GenBank:AY114161], He/80/FR [GenBank:AJ311522], He/80 [GenBank:L27077], pBRT7-HrBDVc [GenBank:AY705791], No/98 [GenBank:AJ311524], ABV [GenBank:EU781967]. Click here for file

Additional file 2 **Alignment of transcription initiation and termination sites in bornavirus genomes**. Panel A, alignment of the 3 bornavirus transcription initiation sites (TIS) and 6 nucleotides of flanking sequences. Panel B, alignment of the 4 bornavirus transcription termination sites. Source genomes for alignments are shown at left. Black trianges highlight ABV sequences. Click here for file

Additional file 3 **Alignment of splice donor and splice acceptor sequences in bornavirus genomes**. Panel A, alignment of splice donor 1 and splice acceptor 1 sequences; Panel B, alignment of splice donor 2 and splice acceptor 2 sequences; Panel C, alignment of splice acceptor 3 sequences. Source genomes for alignments are shown at left. Click here for file

Additional file 4 **Phylogenetic relationships between sub-genomic loci of ABV and representative BDV genomes**. Neighbor-joining trees generated for the indicated nucleotide sequences of ABV and a representative set of BDV genomes are shown for each ORF in the bornavirus genome. Accession numbers of representative BDV genomes are: Ref/V [GenBank:NC_001607], H1766 [GenBank:AJ311523], He/80 [GenBank:AY705791], No/98 [GenBank:AJ311524]. Click here for file

Additional file 5 **Summary of clinical and molecular data for specimens provided in this study**. Microsoft Excel file containing two spreadsheet (US specimens and Israel specimens) summarizing clinical and epidemiologic information available for each specimen, as well as the associated results from the described microarray/PCR/sequence experiments. Click here for file
