# Supplementary figures and images for: Recovery of divergent avian bornaviruses from cases of proventricular dilatation disease: Identification of a candidate etiologic agent
Source: Virol J. 2008 Jul 31;5:88. doi: 10.1186/1743-422X-5-88 (PMC2546392; doi:10.1186/1743-422X-5-88)

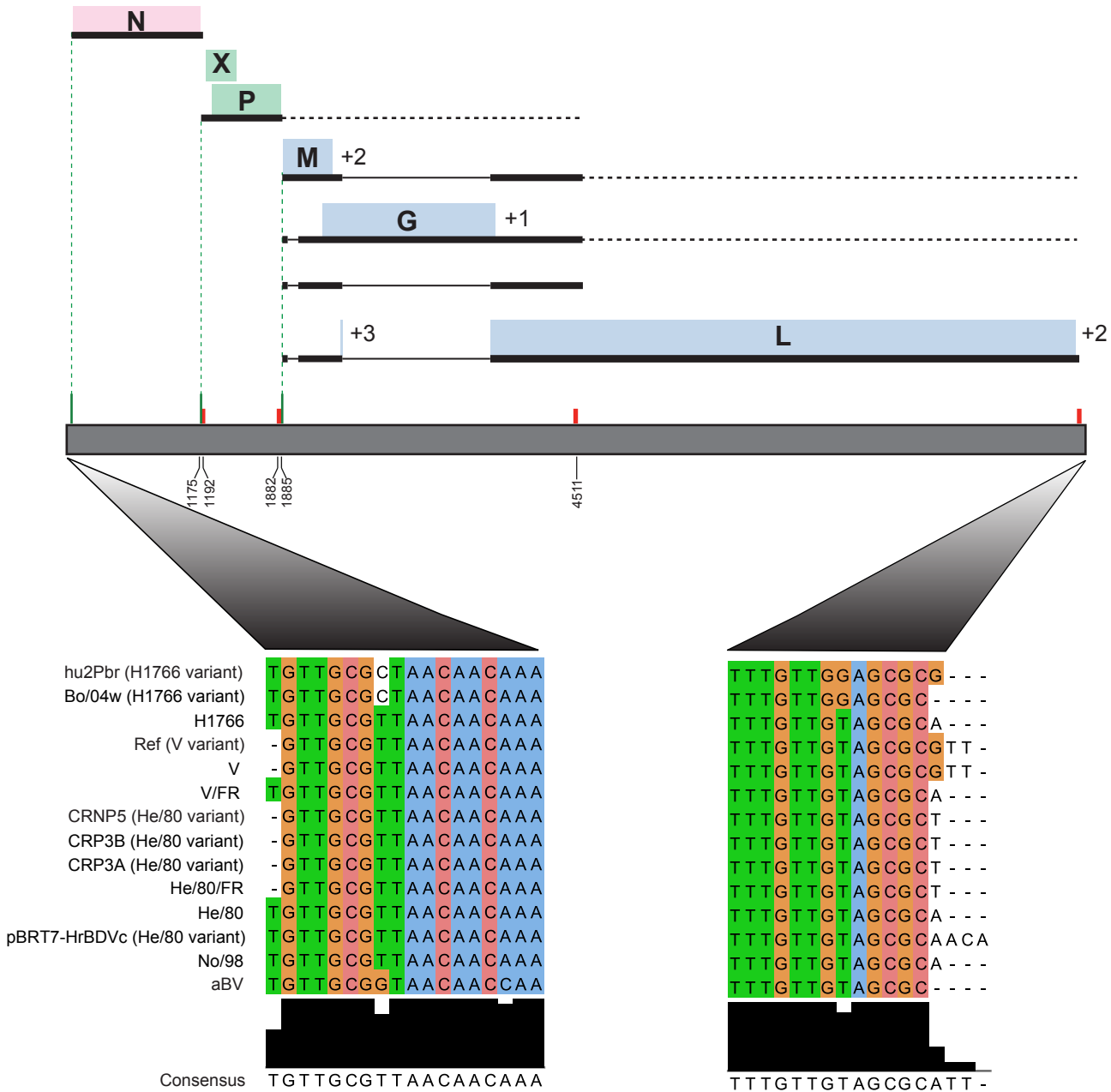

Supplement: Additional file 1 — Alignment of bornavirus genomes 5' and 3' termini. Bornavirus genome organization overview diagrammed as in Figure 2. Sequences in alignments shown are complementary to vRNA sequence, genome isolate names shown at left. 3' end sequence recovered for ABV genome and other BDV genomes is shown in left panel, 5' end sequence recovered for ABV genome and other BDV genomes is shown in right panel. Accession numbers for genomes aligned: hu2Pbr [GenBank:AB258389], Bo/04w [GenBank:AB246670], H1766 [GenBank:AJ311523], Ref [GenBank:NC_001607], V [GenBank:U04608], V/FR [GenBank:AJ311521], CRNP5 [GenBank:AY114163], CRP3B [GenBank:AY114162], CRP3A [GenBank:AY114161], He/80/FR [GenBank:AJ311522], He/80 [GenBank:L27077], pBRT7-HrBDVc [GenBank:AY705791], No/98 [GenBank:AJ311524], ABV [GenBank:EU781967]. [file 1743-422X-5-88-S1.pdf]

A

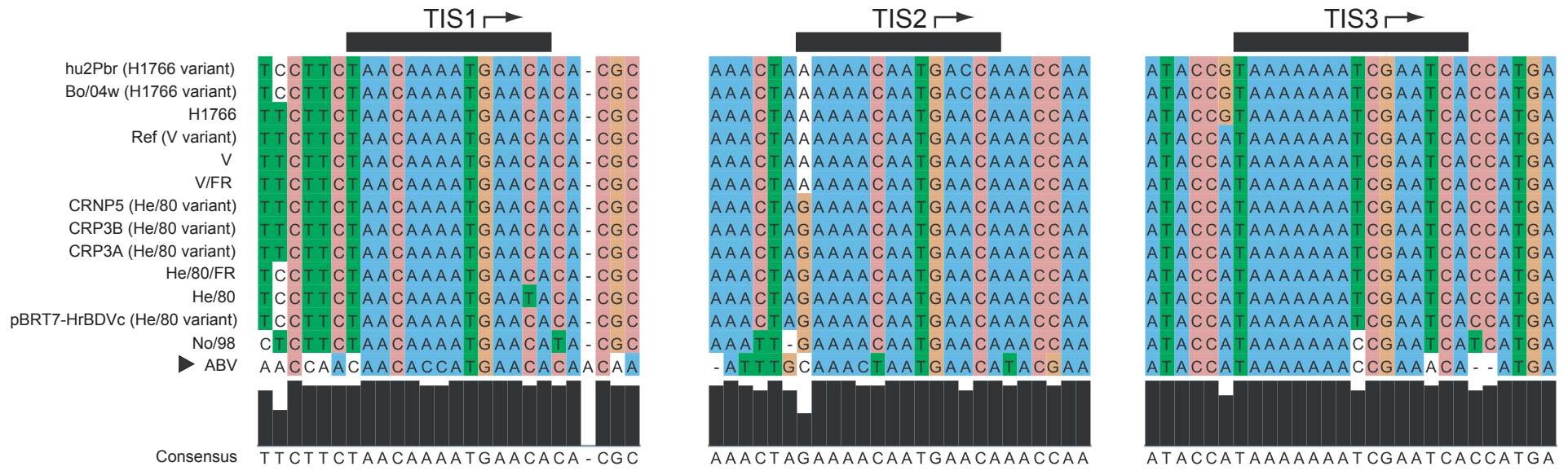

B

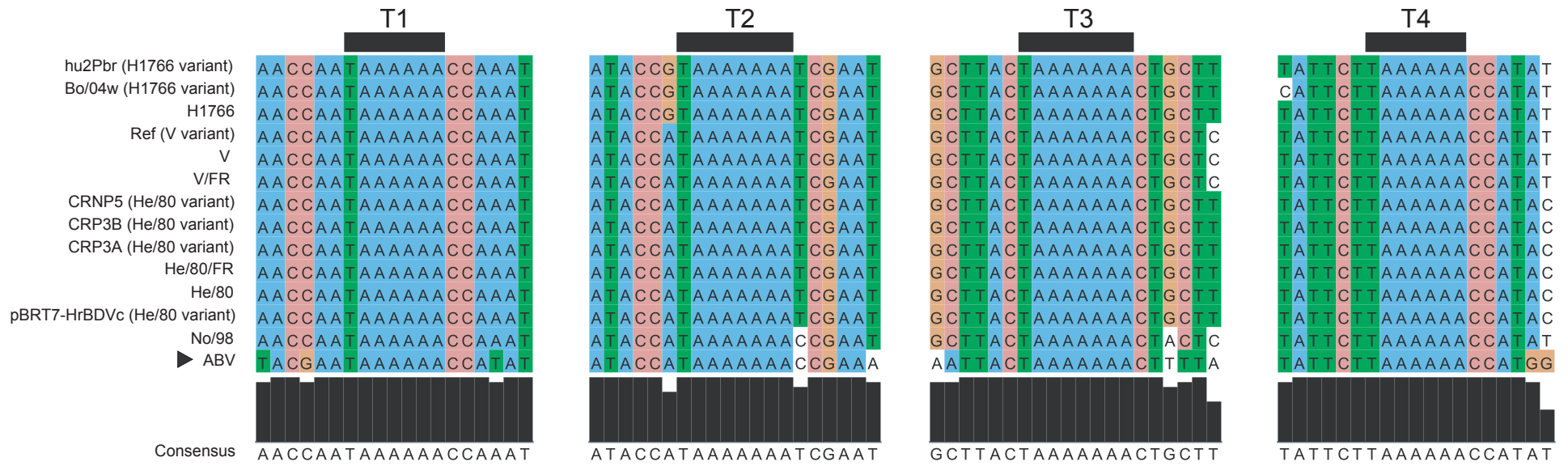

Supplement: Additional file 2 — Alignment of transcription initiation and termination sites in bornavirus genomes. Panel A, alignment of the 3 bornavirus transcription initiation sites (TIS) and 6 nucleotides of flanking sequences. Panel B, alignment of the 4 bornavirus transcription termination sites. Source genomes for alignments are shown at left. Black trianges highlight ABV sequences. [file 1743-422X-5-88-S2.pdf]

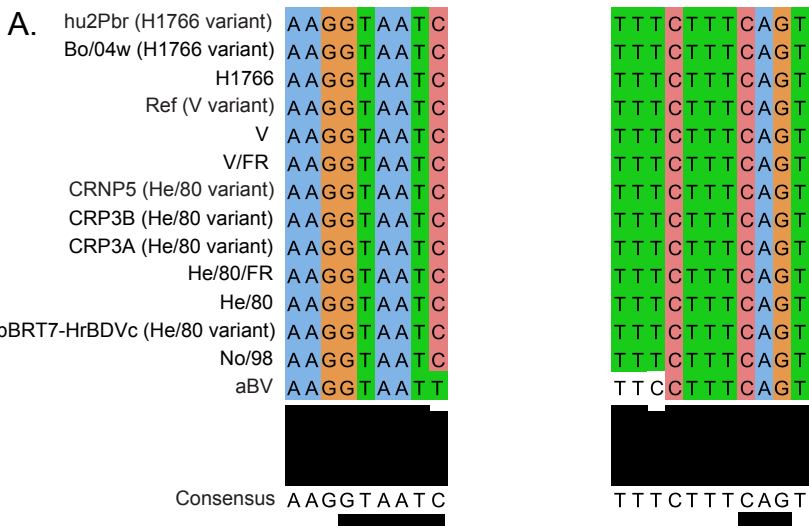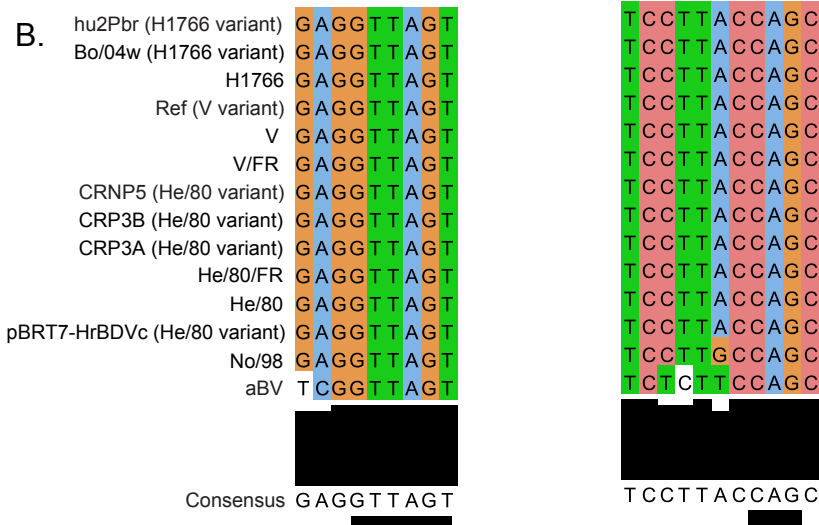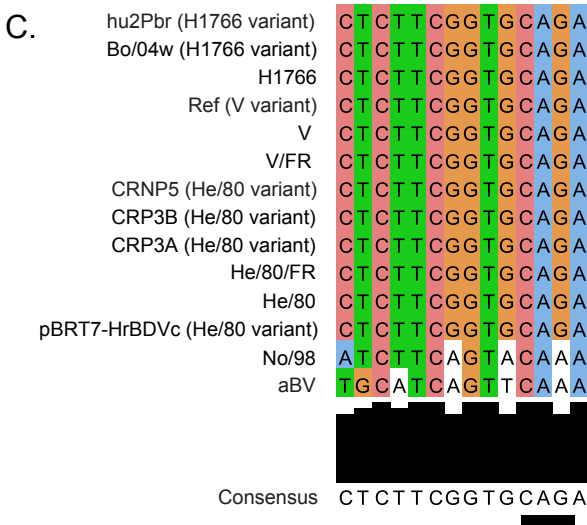

Supplement: Additional file 3 — Alignment of splice donor and splice acceptor sequences in bornavirus genomes. Panel A, alignment of splice donor 1 and splice acceptor 1 sequences; Panel B, alignment of splice donor 2 and splice acceptor 2 sequences; Panel C, alignment of splice acceptor 3 sequences. Source genomes for alignments are shown at left. [file 1743-422X-5-88-S3.pdf]

**N gene**

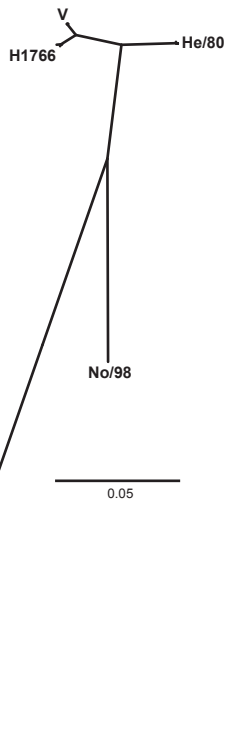

**X gene**

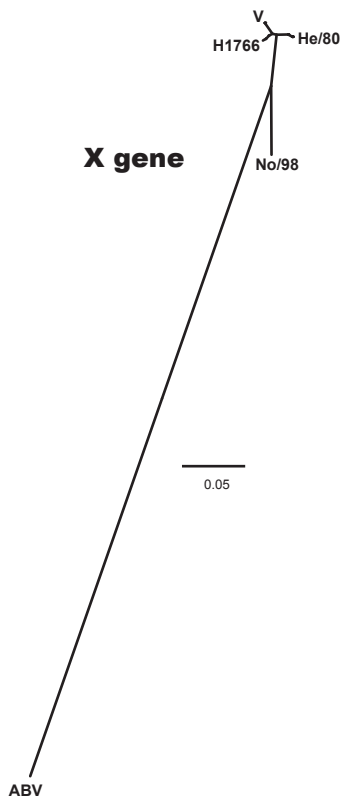

**P gene**

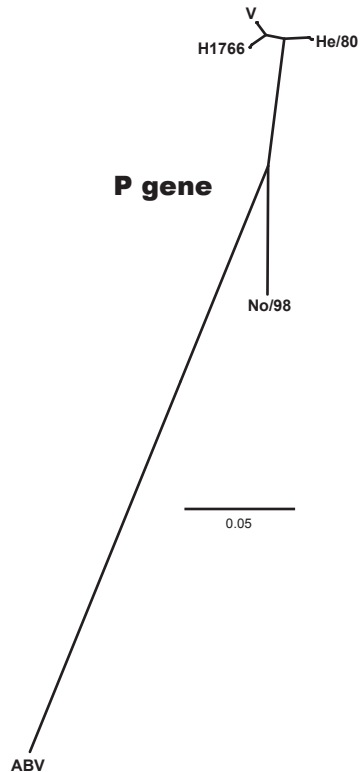

**M gene**

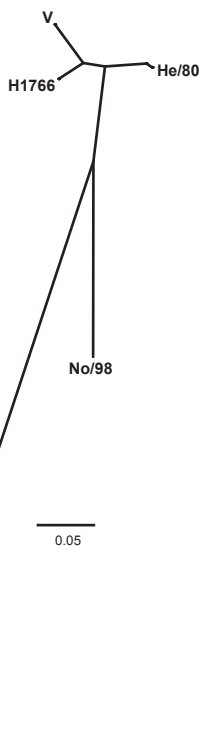

**G gene**

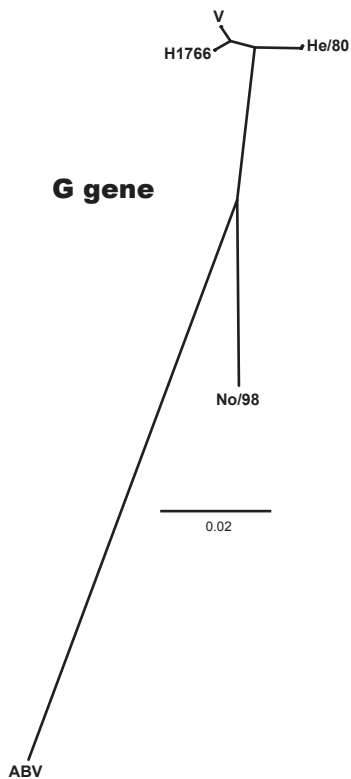

**L gene**

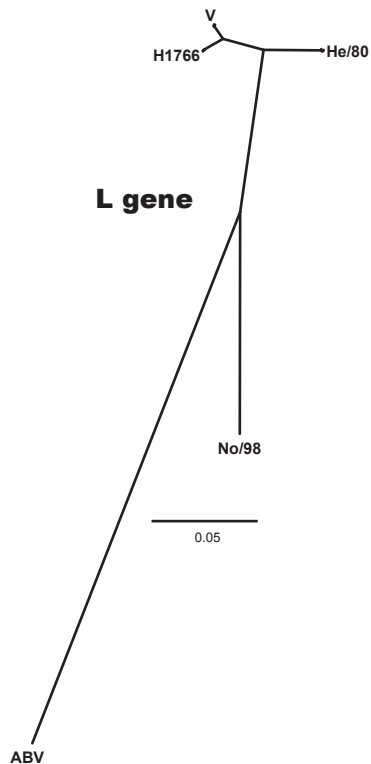

Supplement: Additional file 4 — Phylogenetic relationships between sub-genomic loci of ABV and representative BDV genomes. Neighbor-joining trees generated for the indicated nucleotide sequences of ABV and a representative set of BDV genomes are shown for each ORF in the bornavirus genome. Accession numbers of representative BDV genomes are: Ref/V [GenBank:NC_001607], H1766 [GenBank:AJ311523], He/80 [GenBank:AY705791], No/98 [GenBank:AJ311524]. [file 1743-422X-5-88-S4.pdf]
